# Global Diversity of the Stylasteridae (Cnidaria: Hydrozoa: Athecatae)

**DOI:** 10.1371/journal.pone.0021670

**Published:** 2011-07-22

**Authors:** Stephen D. Cairns

**Affiliations:** Department of Invertebrate Zoology, National Museum of Natural History, Smithsonian Institution, Washington, D.C., United States of America; Heriot-Watt University, United Kingdom

## Abstract

The history and rate of discovery of the 247 valid Recent stylasterid species are discussed and graphed, with emphasis on five historical pulses of species descriptions. A table listing all genera, their species numbers, and their bathymetric ranges are presented. The number of species in 19 oceanographic regions is mapped, the southwestern temperate Pacific (region including New Zealand) having the most species; species are cosmopolitan from the Arctic Circle to the Antarctic at depths from 0 to 2789 m. The current phylogenetic classification of the genera is briefly discussed. An illustrated glossary of 53 morphological characters is presented. Biological and ecological information pertaining to reproduction, development, commensals, and distribution is discussed. Aspects of stylasterid mineralogy and taxa of commercial value are discussed, concluding with suggestions for future work.

## Introduction

Stylasterids, common name “lace corals”, are fragile, usually small, uniplanar to slightly arborescent colonial hydrozoans of the phylum Cnidaria. Their calcium carbonate skeleton is often pigmented orange, red, pink, blue, brown, or violet). The Stylasteridae is the second most species-rich among the 77 hydrozoan families [1], [2], consisting or 247 valid Recent species and an additional 21 exclusively fossil (Paleocene to Recent) species, or a total of 268 valid species. Another 20 species of calcified hydrozoans are known from the families Milleporidae and Hydractiniidae. The stylasterids are known from Antarctica to the Arctic Circle at depths of 0–2789 m, although they are most common at 200–400 m in insular environments; 90% of the species occur deeper than 50 m [3]. Although of limited value in the jewelry trade, stylasterids are important constituents of deep-water coral banks or “coral gardens,” which form substrate for fish and other invertebrates. Following is a brief review of what is known about this fascinating coral family.

Museum abbreviations used in text: BM – The Natural History Museum, London; MNHNP – Muséum National d'Histoire Naturelle, Paris; RMNH – Rijksmuseum van Natuurlijke Historie, Leiden ( = Netherlands Centre for Biodiversity, Naturalis); RSMAS – Rosenstiel School of Marine and Atmospheric Science, Miami; USNM – National Museum of Natural History, Washington DC; WAM – Western Australian Museum, Perth; ZIZM – Zoologische Institut und Zoologisches Musuem, Hamburg; ZSM – Zoologisches Staatssammlung, München.

## Results and Discussion

### History and rate of discovery of Recent species

**Figure 1:**
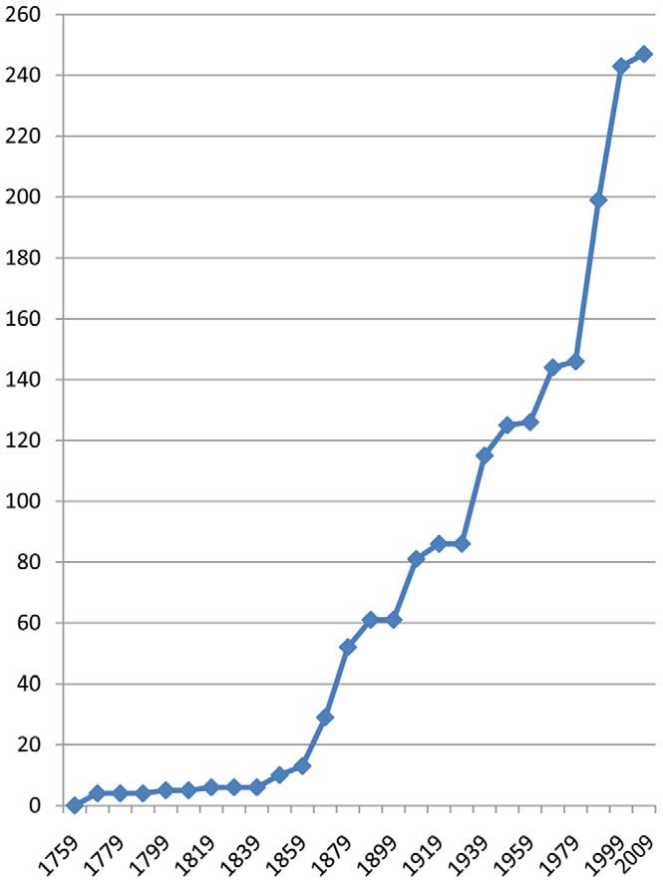
Species accumulation curve of the valid stylasterid species, showing the five pulses of activity.

The first stylasterids described were *Stylaster roseus* (Pallas, 1766) and *Distichopora violacea* (Pallas, 1766) [4], not surprisingly the two most common shallow-water species known from the Atlantic and Pacific Oceans, respectively. The first century post-Linnaeus (1758–1859) was a time of very little interest in this taxon, resulting in only 13 valid species descriptions. The remaining history of species discovery and description is a stepwise progression, the increases correlated to the unpredictable availability of specimens and the availability of people to work on them. Five such steps, or pulses, of species descriptions are discussed below (Figure 1).

**Figure 2:**
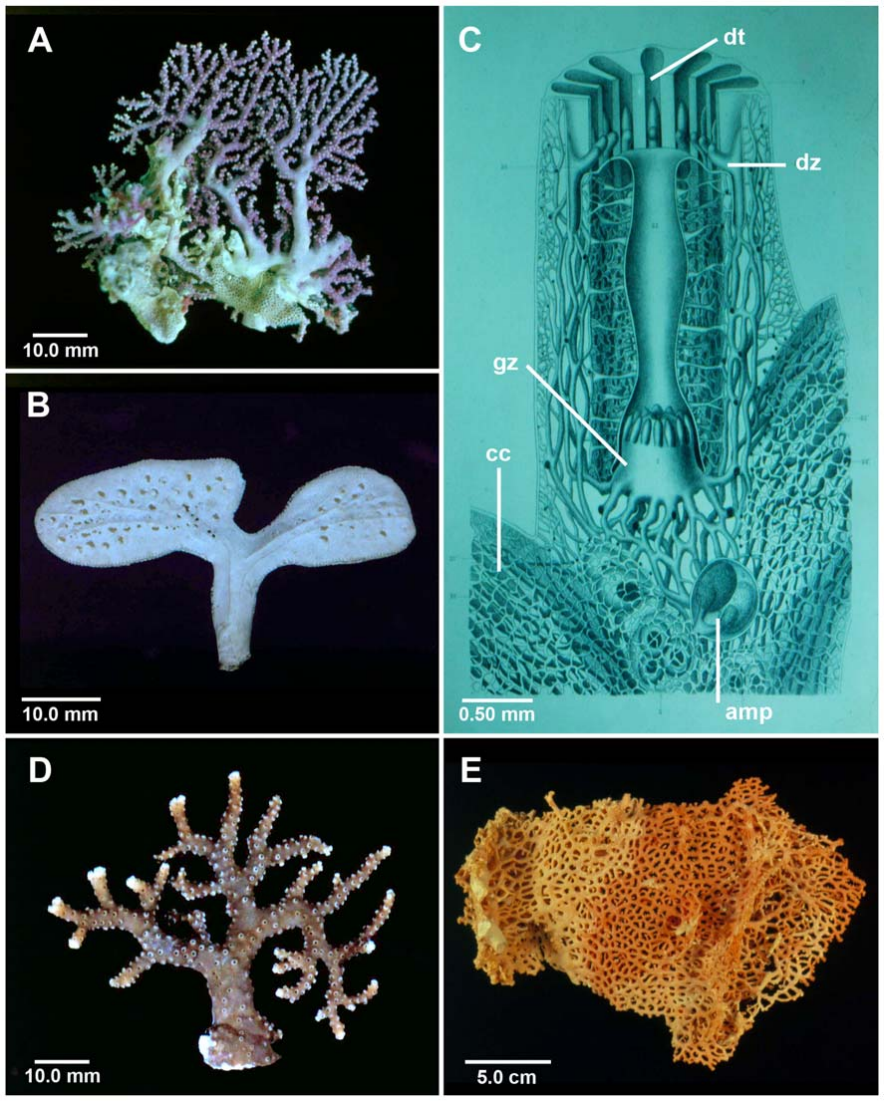
Corallum Shape and Soft Part Anatomy. (A) *Stylaster roseus*, RSMAS, typical branching colony shape, (B) *Distichopora anceps*, USNM 56338, lamellar colony shape, (C) Decalcified corallum of *Stylaster profundus* from Moseley (plate 6 of [13]], showing gastrozooids (gz), dactylozooids (dz), dactylotomes (dt), male gonozooids (gz), and coenosteal canals (cc), (D) *Stylaster brunneus*, WAM 551-87, unusual brown coloration of corallum, (E) *Errinopsis reticulum*, ZIZM, sieve-like reticulum corallum.

The first pulse of species descriptions occurred over an 18-year period (1867–1884), and was fueled primarily by two authors, Pourtalès and Moseley, both of whom relied on collections obtained from deep water, a newly discovered realm of biodiversity and one that stylasterids have heavily exploited. In a series of five papers [5]–[9] Pourtalès described 17 valid species and two valid genera primarily from deep water of the northwestern Atlantic. Pourtalès worked at the Museum of Comparative Zoology at Harvard (Cambridge), and most of his types are still deposited there (see [10]). Moseley [11]–[13], on the other hand, worked on the specimens collected by *HMS Challenger*, which made collections around the world. He described nine valid species and two valid genera, the types of which are deposited at The Natural History Museum (London). Moseley ([12], reprinted as [13]) must also be credited with executing a series of seven exquisite, three-dimensional drawings showing the relationship of the soft parts to the skeleton, “views” that are only now being approximated by means of X-ray computed microtomography [14] have never been equaled since (Figure 2C).

The second small but significant step occurred between 1905–1909 and is attributable to the team of Hickson and England [15], [16], who described 16 valid new species from the Dutch Indonesian *Siboga* Expedition [15] and a collection predominantly from the southwestern Indian Ocean [16]. The *Siboga* types are deposited at the Netherlands Centre for Biodiversity, Naturalis (previously at the ZMA), and the Indian Ocean types at The Natural History Museum, London. Altogether, Hickson described 21 new species (16 co-authored with England) and one new genus.

The third significant increase in species descriptions resulted from the efforts of two men, Fisher and Broch, between 1932 and 1947. Fisher [17], [18] described 15 valid species and two genera primarily from deep water off the Aleutian Islands, collected by the *Albatross*, the types deposited at the National Museum of Natural History, Smithsonian Institution, Washington DC. Broch's [19], [20] “Investigations on Stylasteridae, parts 1 and 2,” described a total of 24 new species and three new genera, primarily from Mortensen's Pacific (1914–1916) and South African (1929–1930) expeditions and Bock's Pacific Expedition (1917), these types deposited at the Zoologisk Museum Copenhagen; he also described three species from the *John Murray* Expedition from the Indian Ocean [21].

The fourth pulse of descriptions was caused by Boschma and Eguchi between 1960 and 1968. Although Boschma published 65 papers on stylasterids from 1951–1970 (see [22] and [23] for a listing of his papers, his new taxa, and a biography], most of his 10 new species and two new genera fall between those years. He studied stylasterids from all parts of the world, most of his types being deposited at the RMNH (now Netherlands Centre for Biodiversity, Naturalis) in Leiden. In my opinion, his most significant contribution, in which he described no new species, was “List of the described species of the order Stylasterina” [24], in which he listed every reference and locality record for every species known to that time. During this time frame Eguchi published three papers in which he described six new species from Japan and Antarctica, his most significant paper being on the stylasterids of Sagami Bay [25].

The last increase in species descriptions correlated to the early career of Cairns (1978–1992), who described a total of 100 new species (eight co-authored) and seven new genera. Some of these were faunistic revisions based on deep-sea expeditions (including the *Albatross*, United States Antarctic Research Program vessels, and vessels associated with the New Zealand National Institute of Water Research and Atmospheric Research, Western Australian Museum, and Rosenstiel School of Marine and Atmospheric Science, University of Miami), such as the revision of the stylasterids from the Antarctic [26], Galápagos [27], [28], western Atlantic [10], New Zealand [29], and eastern Atlantic [30]. Types for most of his new taxa are deposited at the NMNH. Also of note is a description of all genera and their type species [31], the first phylogenetic analysis of the genera [32], tabular and dichotomous keys to the genera [33], and a list of all known species with coarse distribution data [34].

As of 2010, a total of 247 valid species and 26 valid genera had been described, not including 21 exclusively fossil species and one exclusively fossil genus. Lindner et al. (see table S2 in [35]) lists the fossil taxa. Also not included in this total are seven subspecies, three forms, five “facies,” 58 junior synonyms, four *nomina nudae*, two junior homonyms, and six species described without name. All of these names, except for the species described without names, are documented on the WoRMS database (www.marinespecies.org). The stylasterids account for 7.6% of all hydrozoan species and their species accumulation curve (Figure 1) is similar to that of other hydrozoan groups [36]. Eight authors (all mentioned above) account for two-thirds of the described species, and the synonymy rate (number of junior synonyms/total number of described species) is 19%. A list of the 26 valid Recent genera, the number of species contained in each, and their bathymetric ranges are given in Table 1.

**Table 1:** Valid Recent stylasterid genera, numbers of Recent species in each genus, and bathymetric range.

Genus and Author	Number of Species	Bathymetric Range (m)
*Adelopora* Cairns, 1982	4	282–1169
*Astya* Stechow, 1921	2	590–914
*Calyptopora* Boschma, 1968	2	260–2100
*Cheiloporidion* Cairns, 1983	1	642–1137
*Conopora* Moseley, 1879	9	110–2355
*Crypthelia* Milne Edwards & Haime, 1849	31	128–2789
*Cyclohelia* Cairns, 1991	1	27–567
*Distichopora* Lamarck, 1816	25	1–806
*Errina* Gray, 1835	26	6–1772
*Errinopora* Fisher, 1931	6	40–658
*Errinopsis* Broch, 1951	2	250–771
*Gyropora* Boschma, 1960	1	22
*Inferiolabiata* Broch, 1951	3	87–2100
*Lepidopora* Pourtalès, 1871	16	60–2320
*Lepidotheca* Cairns, 1983	13	85–2100
*Paraerrina* Broch, 1942	1	238–274
*Phlangopora* Kirkpatrick, 1887	1	238–274
*Pliobothrus* Pourtalès, 1868	6	80–1600
*Pseudocrypthelia* Cairns, 1983	1	1089
*Sporadopora* Moseley, 1879	3	119–1498
*Stellapora* Cairns, 1983	1	205–1647
*Stenohelia* Kent, 1870	10	91–2021
*Stephanohelia* Cairns, 1991	1	318–793
*Stylantheca* Fisher, 1931	3	0–27
*Stylaster* Gray, 1831	77	0–1485
*Systemapora* Cairns, 1991	1	310–475
Total	247	0–2789

### Biogeography

**Figure 3:**
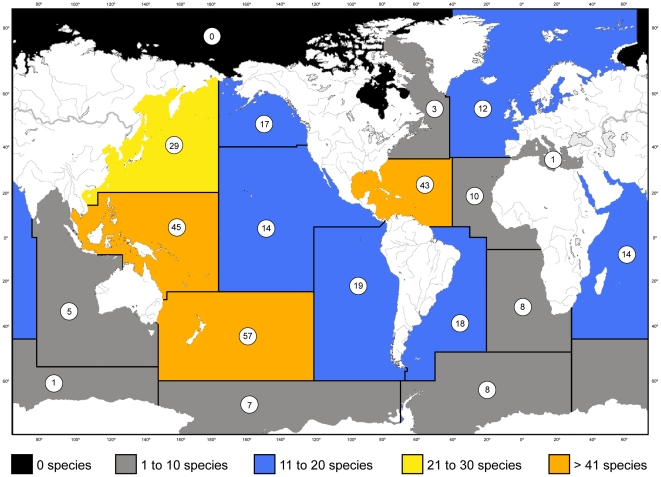
Number of stylasterid species that occur in the 19 FAO oceanic regions.

Using the 19 FAO marine regions (Figure 3) and compilations made by Kitahara [37] as a starting point, the most diverse region for stylasterids is the temperate southwest Pacific (a region including New Zealand), which hosts 57 species, followed by the adjacent tropical southwest Pacific (a region including Indonesia, the Philippines, and New Caledonia), which has 45 species (Figure 3). Together with the northwest Pacific region (including Japan), the western Pacific, in general, is the most diverse oceanic realm for stylasterids. Known but unpublished collections from New Caledonia will probably eventually make the tropical southwest Pacific region the most diverse. A secondary center of diversity is present in the tropical northwest Atlantic (a region including the Caribbean), which hosts 42 species. It is also interesting to note that there are no stylasterids in the Arctic region, and only one species is known from the Antarctic sector of the Indian Ocean, and one from the Mediterranean. The northernmost records from the Pacific are from 58°17′ in the Gulf of Alaska [18], off Pribilof Island in the Bering Sea [38], [39], and the Sea of Okhotsk [40], [41]. Stylasterids are circumpolar in the Antarctic [26], [42]. The stylasterid biodiversity hot spots are not unlike that of deep-sea Scleractinia (Figure 3 in [3]). Using 200 m intervals, Kitahara (Figure 19 in [37]) also tabulated that the depth range of 200–400 m was most common for stylasterids, a finding consistent with that of Cairns [10], [29] based on regional revisions. Cairns [3] tabulated that 90% of the species occurred at depths greater than 50 m, the deepest species, *Crypthelia affinis* Moseley, 1879, occurring at 2789 m [30].

Using a phylogeny based on molecular data, Lindner et al. [35] suggested that the stylasterids originated in deep water and subsequently invaded shallow water at least four times, twice within the genus *Stylaster* and twice within the genus *Distichopora*. This offshore-onshore scenario is contrary to the more commonly held concept of an onshore-offshore pattern for most marine groups (Jablonski et al., 1983).

### Classification and phylogeny

Although originally considered to be a hydroid family [11], [12], the stylasterids were later considered to be an order of hydroids [43], but are now once more considered to be only a large and highly diverse family, the Stylasteridae, one of five families in the superfamily Hydractinoidea within the order Filifera (species having filiform tentacles) [1]. Milleporidae was also considered as an order, but now as a family in the order Capitata (species having capitate tentacles). Thus the old designation of “Hydrocorallia” that referred to the two calcified hydroid families, the stylasterids and milleporids, must be abandoned since it includes families in two different orders linked only by the convergent character of a calcified skeleton.

**Figure 4:**
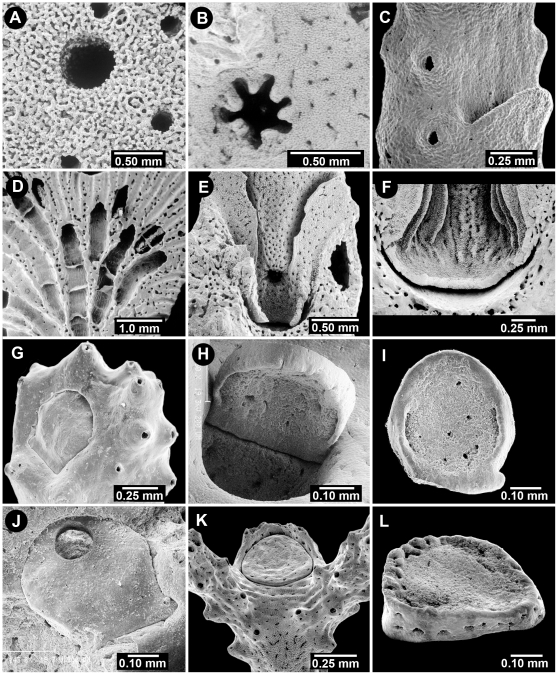
Aspects of the Gastropore and Gastropore Tube. (A) *Sporadopora dichotoma*, USNM 52647, a flush gastropore and several smaller flush dactylopores, (B) *Stellapora echinata*, USNM 59945, a stellate gastropore, (C) *Lepidopora* sp., BM 1890.4.11.24, a broad abcauline gastropore lip, (D) *Distichopora uniserialis*, USNM 15969, horizontal tabulae in axial gastropores, (E) *Crypthelia formosa*, USNM 60084, double-chambered gastropore tube, female ampullae in ring around cyclosystem with an open efferent pore at base of lid, (F) *Crypthelia robusta*, NZOI P-919, double-chambered gastropore tube showing ring constriction, (G–I) *Adelopora pseudothyron*, USNM 60128, gastropore operculum showing the closed type of opercular articulation, in the closed and open position, and underside of one showing an articulating nub, respectively, (J) Same as G–I, an operculum bored by a predator, (K–L) *Adelopora fragilis*, MNHNP, an example of the open opercular articulation.

**Figure 5:**
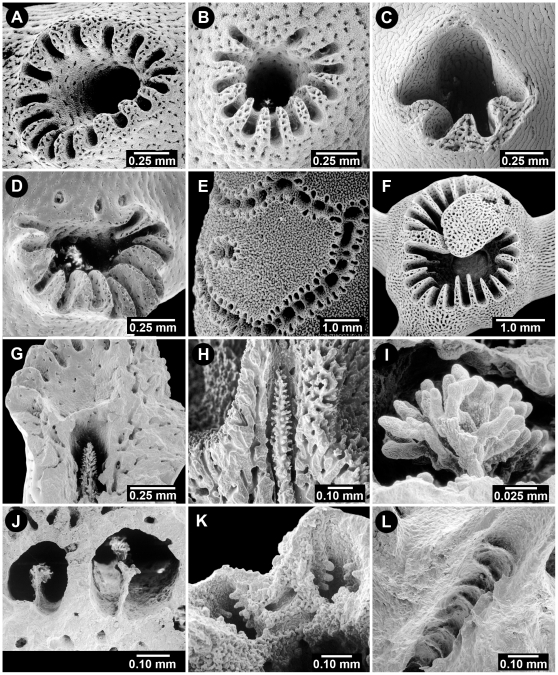
Aspects of the Cyclosystem and Dactylostyles. (A) *Stylaster galapagensis*, USNM 72099, a typical cyclosystem, (B) *Stylaster roseus*, USNM 47807, a cyclosystem with a narrow adcauline diastema, (C) *Conopora tetrastichopora*, USNM 87566, a cyclosystem with a wide adcauline diastema, (D) *Stylaster atlanticus*, USNM 71824, a cyclosystem with some obsolete adcauline dactylopores, (E) *Distichopora vervoorti*, RMNH 23976, distichoporine arrangement of pores, (F) *Crypthelia dactylopoma*, USNM 72110, a cyclosystem with a narrow fixed lid, (G) *Stylaster laevigatus*, USNM 71798, gastropore shelf, (H) *Errinopora pourtalesii*, USNM 52254, a robust dactylostyle, (I) *Stylaster miniatus*, USNM 72151, a robust dactylostyle in apical view, (J) *Distichopora dispar*, USNM 85116, dactyloridges, (K) *Inferiolabiata labiata*, USNM 59951, lateral dactylostyles, (L) *Lepidotheca robusta*, USNM 85106, pseudotabulae in a dactylopore spine.

**Figure 6:**
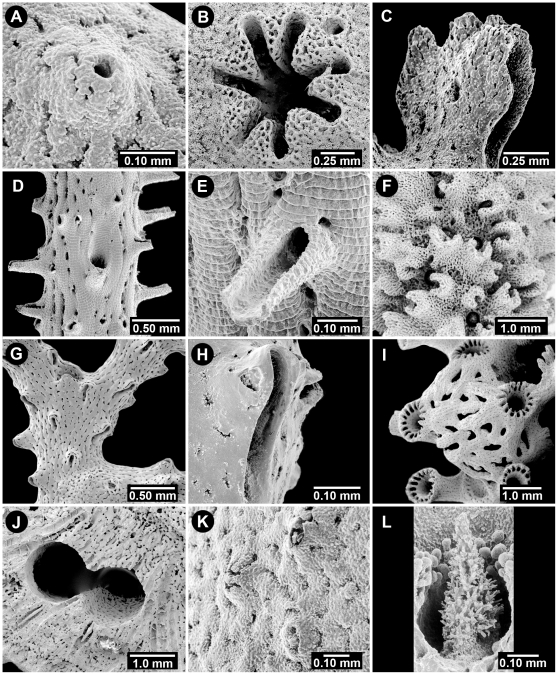
Aspects of Dactylopores, Dactylopore Spines, and Miscellany. (A) *Lepidopora sarmentosa*, USNM 60135, a conical dactylopore mound, (B) *Stylaster stejnegeri*, USNM 43271, a cyclosystem with a supernumerary dactylopore, (C) *Errina gracilis*, USNM 60242, composite dactylopore spine, (D–E) *Lepidotheca altispina*, USNM 85103, tall cylindrical abcauline dactylopore spines and reverse polarity platelets, (F) *Errinopora pourtalesii*, USNM 52254, compound dactylopore spines, (G) *Errina dendyi*, USNM 76302, adcauline dactylopore spines, (H) *Errina cheilopora*, USNM 85134, adcauline dactylopore spine, (I) *Stenohelia concinna*, USNM 84747, polychaete gall, (J) *Distichopora robusta*, USNM 1020571, double axial tube formed by *Polydora* polychaete, (K) *Calyptopora reticulata*, USNM 60010, coenosteal papillae, (L) *Stenohelia profunda*, USNM 52244, a robust ring palisade.

The first phylogenetic analysis of the stylasterid genera was published by Cairns [32] and later elaborated upon by him [44], both based entirely on gross skeletal morphology. The fossil genus *Axopora*, or an axoporid-like ancestor, was considered to be the proximate ancestor, in the late Cretaceous (Maastrichtian) [45], and a linear sequence of evolutionary adaptations was suggested leading from simple to complex morphology. Three trends were suggested: 1) the development of a double-chambered, lidded gastropore tube (Figures 4E–F, 5F), with a concomitant loss of gastro- and dactylostyles, and gastrozooid tentacles, 2) structural modification and orientation of the dactylopore spine (Figures 6C–G), and 3) progressive coordination of all three polyp types from a randomly arranged configuration to a well-coordinated functional unit – the cyclosystem (Figures 5A–D). All three trends were hypothesized to have improved feeding efficiency and improved the defense of its gastro- and gonozooids. The only other phylogenetic analysis was published by Lindner et al. [35], and was based on molecular sequences of three genes (one mitochondrial and two nuclear). This phylogenetic tree is quite different, suggesting three discrete clades: one leads to the double-chambered genera via *Conopora*, *Pliobothrus* and *Lepidopora microstylus*, a second includes most of the species of *Stylaster*, and a third includes all the other genera, with equivocal ancestry. Thus all three clades may have a cyclosystemate ancestor, contrary to the scenario of Cairns. Obviously, more genes will have to be sequenced, the morphology must be integrated into the analysis, and the results more fully scrutinized.

### Anatomical glossary

**Figure 7:**
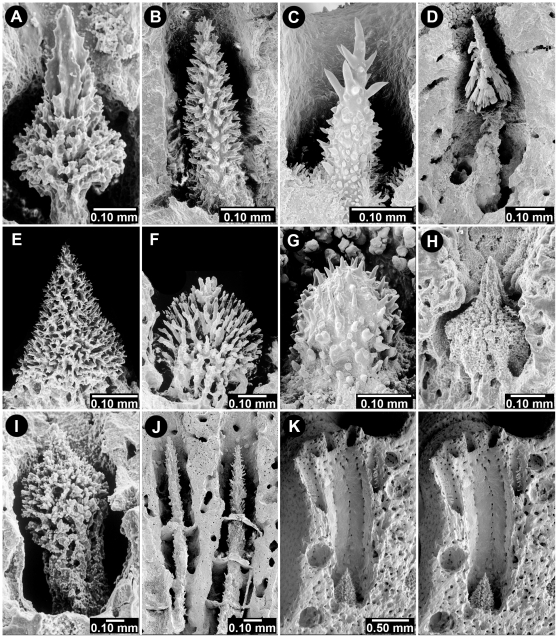
Various Gastrostyle Shapes. (A) *Errinopsis fenestrata*, USNM 52694, (B) *Stylaster corallium*, USNM 71829, (C) *Systemapora ornata*, USNM 85117, (D) *Lepidopora dendrostylus*, USNM 60251, (E) *Stylaster profundus*, BM 1880.11.25.174, (F) *Stylaster erubescens meteorensis*, ZSM, (G) *Calyptopora sinuosa*, USNM 87536, (H) *Cheiloporidion pulvinatum*, USNM 52648, (I) *Lepidopora sarmentosa*, USNM 60135, (J) *Distichopora robusta*, USNM1020570, needle-shaped gastrostyles stabilized by tabulae, (K) *Stylaster eguchii*, USNM 85143, stereo view showing a deep gastropore tube, gastrostyle, internal male ampullae, and a dactylostyle (upper right).

The only glossary for the group was published by Fisher (1938), who included 16 terms and was published long before the advent of scanning electron microscopy (SEM). SEM of stylasterids was first published by Sorauf [46] and Fenniger and Flajs [47] to illustrate microstructure, but not used for taxonomic purposes until 1982 [48] and then routinely thereafter. Stereo SEM views (Figure 7K) are also used effectively to show the inter-relationships of various skeletal characters. Many new structures were discovered and named using SEM but their definitions were never consolidated, thus motivating the following illustrated glossary. Many of the characters defined below were originally described or discussed by Boschma [43] and Cairns [10], [11], [26], [27], [29].

### Glossary

**Abcauline and Adcauline Dactylopore Spine**: See Dactylopore Spine.

**Figure 8:**
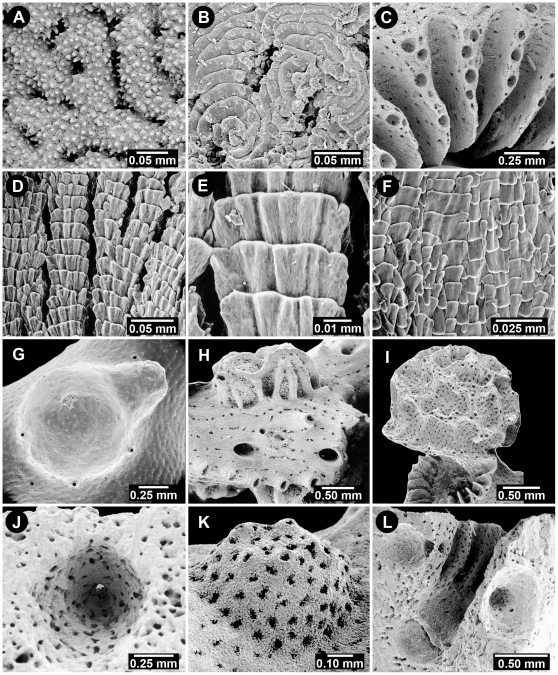
Aspects of Coenosteal Texture, Pseudosepta, and Ampullae. (A) *Stylaster verrillii*, USNM 1123299, reticulate granular coenosteal texture, (B) *Errina sinuosa*, USNM 85131, radial-imbricate coenosteal texture, (C) *Crypthelia trophostega*, USNM 1122887, nematopores on thin pseudosepta, (D–E) *Errina altispina*, USNM 71778, linear-imbricate coenosteal texture (normal polarity), (F) *Systemapora ornata*, USNM 85117, contiguous alternating polarity of imbricating platelets, (G) *Adelopora pseudothyron*, USNM 60128, superficial female ampulla with a large lateral efferent tube, (H) *Distichopora anomala*, USNM 71813, stellate-ridged superficial female ampullae, (I) *Crypthelia lacunosa*, USNM 45684, large female ampulla in cyclosystem lid, itself covered with reticulate ridges and nematopores, (J) *Sporadopora dichotoma*, USNM 60100, internal female ampulla with a small efferent duct to surface, (K) *Stenohelia robusta*, USNM 21283, cluster of superficial male ampullae with small apical efferent pores, (L) *Conopora pauciseptata*, USNM 52619, internal male ampullae flanking a double-chambered gastropore tube.

**Ampulla (Ampullae)**: The skeletal encasement of the gonophore, often forming prominent hemispherical (Figures 8G, I, K) or more rarely stellate-shaped (Figure 8H) **superficial** blisters on the coenosteum or forming spherical **internal** (Figures 7K, 8J, L) chambers beneath the coenosteal surface. Superficial female ampullae release their planulae through a lateral **efferent pore** or **tube** (Figure 8G), whereas the smaller male ampullae usually release their sperm through much smaller multiple apical pores (Figure 8K). Internal ampullae release their gametes through **efferent ducts** (Figure 8J) that communicate directly to the coenosteal surface, or in some cases, through pseudosepta or beneath cyclosystem lids (Figure 4E). The methods of gamete and planular release are so varied in the genus *Crypthelia*, that an **ampullar formula** was devised by Cairns [27], which is a combination of the three described variations of larval release and eight variations of sperm release.

**Ampullar Formula**: See Ampulla.

**Axial Gastropore Tube**: See Gastropore Tube.

**Cheval-de-frise**: See Gastropore Tube.

**Coenosarc**: The network of canals that connect the polyps (Figure 2C).

**Coenosteum (Coenosteal**, adj.): The calcium carbonate skeleton of the stylasterid, usually aragonitic, but occasionally calcitic or partially calcitic [49]. The branch microstructure is usually composed of narrow (50–80 µm wide) **strips** (Figure 8D, E) of coenosteum which are separated by thin (5–10 µm wide) **slits** (Figure 8D, E) or series of pores, which allow communication of the coenosteal canals (Figure 2C) from the interior to the exterior [26], [31]. If the strips are arranged in an irregular reticulate fashion and the surface of the strips is covered with small granules, the texture is termed **reticulate-granular** (Figure 8A). If the strips are parallel, longitudinal, and covered with imbricating platelets (Figures 8E; also see **Platelet**), it is termed **linear-imbricate** (Figures 8D–F, also see [31]). **Radial-imbricate** (Figure 8B) arrangements have also been observed.

**Composite Dactylopore Spine**: See Dactylopore Spine.

**Compound Dactylopore Spine**: See Dactylopore Spine.

**Corallum**: The calcareous skeleton of the entire colony.

**Cyclosystem (Cyclosystemate**, adj.): A functional unit of stylasterid colony structure composed of a central gastropore (gastrozooid) surrounded by a variable number of dactylopores (dactylozooids) (Figures 5A–D, F). Occasionally dactylopores on the adcauline side become infilled or fail to develop, resulting in a gap or hiatus in this region, called a **diastema** (Figures 5B–D). Other arrangements of polyp types include distichoporine and random. Several genera have fixed, horizontal to oblique **lids** (Figures 5F, 8I) derived from the enlargement of one or more abcauline pseudodsepta that cover part or all of the cyclosystem.

**Dactylopore**: The surface pore associated with a dactylozooid, usually round or elliptical in shape, and flush (Figure 4A) or slightly mounded (Figure 6A). In cyclosystemate species, dactylopores occasionally occur randomly, in addition to the cyclosystem configuration, these isolated dactylopores referred to as **supernumerary** (Figure 6B). Incomplete tabulae that do not completely wall off a section of dactylopore tube are called **pseudotabulae** (Figure 5L, also see [29]).

**Dactylopore Spine**: A projection from the coenosteum, U- or horseshoe-shaped in cross section, that is usually adjacent to a dactylopore (Figures 6E–H). If the lateral slit (**dactylotome**) (Figure 2C) of the dactylopore spine is directed toward the distal branch tip, it is termed **abcauline** (Figures 6D–E); if it opens toward the proximal end of the branch, **adcauline** (Figures 6G–H). Some species may have two shapes of dactylopore spines, this condition termed **dimorphic**. Occasionally dactylopore spines are clustered together, their back sides fused to one another in a random orientation, these termed **compound dactylopore spines** (Figure 6F). A **composite dactylopore spine** (Figure 6C) has multiple dactylotomes [31].

**Dactyloridge**: See Dactylostyle.

**Dactylostyle**: A row or crowded multiple rows of small cylindrical pillars (elements) that occur on the outer wall of the dactylopore tube (Figures 5H–I, 7K), In some species two additional rows of elements occur on the two lateral sides of the dactylopore tube, termed **lateral dactylostyles** (Figure 5K, also see [10]). In one species, *Distichopora dispar* Cairns, 1991, the dactylostyle is a solid ridge, termed a **dactyloridge** (Figure 5J, also see [29]).

**Dactylotome**: See Dactylopore Spine.

**Dactylozooid**: One of the three types of stylasterid polyps, which specializes in defense and aiding in food acquisition, each dactylozooid composed of a simple mouthless tentacle (Figure 2C).

**Diastema**: See Cyclosystem.

**Dimorphic Dactylopore Spine**: See Dactylopore Spine.

**Distichoporine**: A functional unit of stylasterid colony structure in which a row of gastropores is flanked on both sides by a row of dactylopores (Figures 5E, 8H). Other arrangements of polyp types include cyclosystemate, linear, unifacial, and random.

**Double-Chamber Gastropore Tube**: See Gastropore Tube.

**Efferent Duct**: See Ampulla.

**Efferent Pore**: See Ampulla.

**Gastropore** (also called **Gastrostome**): The surface pore associated with a gastrozooid, usually round (Figures 4A, 5A–B), slightly elliptical, or stellate (Figure 4B) in shape; occasionally bordered by a triangular, abcauline **lip** (Figure 4C). In the genus *Adelopora*, the gastropore tube is covered by a close-fitting, unattached (movable) **operculum** (Figures 4G–L). If the operculum has hinges that lock into the coenosteum in order to facilitate its movement, it is said to have a **closed opercular articulation** (Figures 4G–J); if the operculum is unhinged and thus not locked into the coenosteum, an **open opercular articulation** (Figures 4K–L, also see [29]).

**Gastropore Ring Constriction**: See Gastropore Tube.

**Gastropore Tube**: The tube that contains the gastrozooid, usually a simply straight cylinder (Figure 7K), but often curved, and in some genera, a **double-chambered** cavity (Figures 4E–F, 8L), the upper and lower chambers separated by a **gastropore ring constriction** (Figure 4F). In some species the tube contains a horizontal platform through which the tube penetrates, the platform called a **shelf** (Figure 5G, also see [27]). Gastropore (and dactylopore) tubes are usually fairly short and oriented perpendicular to the branch surface, termed **peripheral gastropore tubes**, but in some genera they are quite elongate, following the axis of the branch in a cluster for some distance, thus termed **axial gastropore tubes** (Figure 4D). In such long tubes the correspondingly long gastrostyles are stabilized by horizontal plates called **tabulae** (Figures 4D, 7J). Some genera have a ring or girdle of small cylindrical elements that project from the wall of the gastropore tube near the level of the gastrostyle tip, this structure called the **ring palisade** (Figures 6L, 7G, also see [31])(also called the ***cheval-de-frise***). These elements are often the same size and shape as the dactylostyle elements.

**Gastrostome**: See Gastropore.

**Gastrostyle**: The vertical, spinose, axial structure that projects from the base of the gastropore tube in various genera. Although usually lanceolate in shape (Figure 7A), it may assume a variety of other shapes (Figures 7B–K).

**Gastrozooid**: One of the three types of stylasterid polyps, which specializes in obtaining food, and usually containing a ring of filiform tentacles encircling a mouth (Figure 2C).

**Gonophore**: One of the three types of stylasterid polyps, this one housing the reproductive structures, i.e., sperm or egg (Figure 2C). Most species are dioecious, each colony being either male or female, only one species known to be hermaphroditic (*Stylaster roseus*).

**Internal Ampulla**: See Ampulla.

**Lateral Dactylostyle**: See Dactylostyle.

**Lid, Cyclosystem**: See Cyclosystem.

**Linear-Imbricate Coenosteal Texture**: See Coenosteum.

**Lip, Gastropore**: See Gastropore.

**Nematophores**: Concentrations of large nematocysts, often located at the edge of cyclosystems, on pseudosepta, or on cyclosystem lids. **Nematopores** (Figures 8C, I) are the shallow skeletal pits that house the nematophores.

**Nematopores**: See Nematophore.

**Opercular Articulation (Open or Closed)**: See Gastropore.

**Operculum**: See Gastropore.

**Papilla (Papillae)**: Conical, apically perforate nematocyst-bearing structures that occur on the coenosteum of some species (Figure 6K), usually less well defined than nematophores.

**Peripheral Gastropore Tube**: See Gastropore Tube.

**Planula (Planulae)**: The specialized larval stage of a cnidarian. All stylasterids are brooders, releasing their planulae at an advanced stage.

**Platelet**: The branch coenosteum of a species having linear-imbricate texture is composed of imbricating platelets. If the leading edges of the platelets are facing distally toward the branch tip, it is termed **normal polarity** (Figures 8D–E), if facing proximally, then **reverse polarity** (Figures 6D–E), and if both orientations are present in the same specimen, then **alternating polarity** (Figure 8F, also see [29]).

**Polarity (Normal, Reverse, Alternating)**: See Platelet.

**Pseudoseptum (Pseudosepta)**: The roughly triangular-shaped coenosteum that separates the dactylotomes in a cyclosystem (Figure 8C).

**Reticulate-Granular Coenosteal Texture**: See Coenosteum.

**Ring Palisade**: See Gastropore Tube.

**Shelf, Gastropore**: See Gastropore.

**Slit, Coenosteal**: See Coenosteum.

**Strip, Coenosteal**: See Coenosteum.

**Superficial Ampulla**: See Ampulla.

**Supernumerary Dactylopore**: See Dactylopore.

**Tabula (Tabulae)**: See Gastropore Tube.

### Biology, ccology and environment

What little we know about the biology and ecology of stylasterids is limited primarily to shallow-water species, which constitute less than 10% of the total diversity of stylasterids. The classic study of stylasterid natural history was made by G. L. Ostarello [50], [51], who studied the shallow-water *Stylaster californicus* for her PhD dissertation (see also [52] for a population study of New Zealand species). Observing the living animal, she was able to study aspects of commensalism, reproductive cycles, method of fertilization, development and release of the planulae, dispersal, settlement, mortality, and regeneration. She found this species to have a very short planular dispersal stage, generally not entering the plankton for very long, if at all. A number of studies have addressed the early development of the gonophore through the formation of gastrozooids and cyclosystems, some of the more pertinent papers being those of Hickson [53], [54], Goedbloed [55], Frichman [56], Brooke and Stone [57], and Puce et al. [58].

Stylasterid colonies are usually either male or female, rarely hermaphroditic, always with strong sexual dimorphism of the skeletal ampullae, which is often used to help identify species. Once the egg is fertilized, it grows to the advanced planular stage before it is released through an efferent canal, after which it usually crawls away and settles a short distance from the parent. This produces rather limited distributions and high regional endemicity [57]. As an extreme example, *Distichopora anceps*, a distinctively shaped species described by Cairns in 1978 [59] from off Laysan, Hawaiian Islands (Figure 2B), could not be found again in the northwest Hawaiian Islands despite intensive searching by submersible and ROV, until the precise type locality was re-visited where it was found to be the dominant benthic invertebrate in what may be the entire species range of about 10 square km [60].

Stylasterids are host to a number of commensals, the tiny (4–6 mm in greater diameter) ovulid gastropod of the genus *Pedicularia* being an obligate symbiont on various stylasterid species (see [61] for a review). The seven species of this genus are flat and limpet-like in shape, assuming the color of the stylasterid, thus being quite inconspicuous. If the snail becomes detached, a characteristic elliptical scar remains to document the association. Both spionid and polynoid polychaetes are common commensals in some stylasterid species, the former boring parallel, binary tubes along branch axes (see [62] and Figure 6J herein), the latter, and perhaps more common polychaete commensal, forming elongate, cage-like tubes along the stylasterid branches (Figure 6I, also see [63]), through which the worm travels. Other less common commensals include nemerteans, pycnogonid larvae, thoracic and ascothoracic cirripids, barnacles, copepod galls, bryozoans (*Heteropora pacifica*), and cyanobacteria [64], most of these associations briefly reviewed by Zibrowius [65] and Zibrowius and Cairns [30]. Because some stylasterid species can attain a relatively large size (1 m) and occur in high density, they often contribute to the structure of deep-water coral banks, and thus provide habitat for fish [66], [67] and other invertebrates. Roberts, et al. [68] lists 15 such habitat-forming species, some of which are very common in the Aleutian Islands [39], off southern California [67], the Pacific Subantarctic [26], the Blake Plateau [10], and the seamounts off New Zealand [29]. All stylasterids are firmly attached to a substrate except for *Conopora adeta* Cairns, 1987 [69], which is unattached, forming a dense bolus of calcium carbonate around a commensal polychaete worm, which provides a kind of anchor for the colony.

One of the most exotic stylasterids known, *Adelopora pseudothyron* Cairns, 1982 [48], has evolved tightly fitted, hinged and thus moveable lids that cover each gastropore, affording significant protection to the gastrozooid beneath, which in all other species is partially exposed to predation (Figures 4G–I). Cairns [48] hypothesized that this was an evolutionary response to the very competitive environment of the Subantarctic Pacific deep-water bank on which it was found. But, even this bastion of defense has been known to be breached by a predator (?molluscan) that bored a small hole (0.12 mm in diameter) directly through the lid (Figure 4J).

Cairns [70] plotted the distribution of every stylasterid known to that date and found a pronounced insular distribution pattern, species being found primarily off small (less than 36,000 km^2^) oceanic islands, and atolls, and on seamounts and submerged ridges of the appropriate depth, rarely off continental land masses or “high” islands. He hypothesized that the absence of stylasterids from the proximity of continental land masses might be explained by their sensitivity to fluctuating salinity and sedimentation associated with that environment. Also, their predilection for insular distribution might be explained by their need for a hard substrate and their preference for a low nutrient level environment characteristic of a K-strategist, low nutrients being characteristic of most insular environments. Some exceptions to this pattern occur [71], but, in general, it seems to be the common pattern.

Two hundred thirty-nine, or 97% of the stylasterid species, have a branching colony mode (Figures 2A, D); three are encrusting; and five form lamellate sheets. The encrusting species, all in the genus *Stylantheca*, are easy to explain as an adaptation to a high-energy intertidal environment, but the cause of the lamellate growth form (four of the five species occurring in the Aleutian Islands, see [39]) is, as yet, unexplained. Two species also form porous reticulate fans (Figure 2E).

### Mineralogy

All living Scleractinia and most stylasterids (76% according to a study by Cairns and Macintyre, [49]) form skeletons of the aragonitic polymorph of calcium carbonate, the other 24% using the less soluble polymorph calcite or partially calcitic. Six of the seven Aleutian Islands stylasterids analyzed by Cairns and Macintyre [49] were calcitic, an unusually high percentage. Although they did not suggest it at the time, later Cairns [3] and Guinotte, et al. [72] pointed out that the aragonite saturation horizon (ASH) in the North Pacific is quite shallow (less than 150 m), which would mitigate against an aragonitic skeleton but favor a less soluble calcitic skeleton at depths beyond 150 m. More sampling and analysis is needed to substantiate this correlation but the coincidence is striking.

### Human interest

At least one stylasterid species has had some commercial value, the purple California hydrocoral *Stylaster californicus* (Verrill, 1866), known from relatively shallow water (0–110 m) from San Francisco to Baja California. Starting in the early 1970's this species was collected and sold as curios for up to $150 and manufactured into jewelry, a necklace valued at up to $250 [67], [73]. Because of overfishing, the state of California eventually established the Farnsworth Bank Ecological Reserve near Santa Catalina to protect this species. All stylasterids, as well as *Millepora*, were placed on the CITES Appendix II in 1990, which regulates the trade of these taxa across international borders. *Stylaster californicus* has a rather porous corallum, which is not amenable to cutting, carving, and polishing, in contrast to the skeletons of precious and black corals, but many other stylasterids do have a hard and colorful corallum quite similar to that of the precious coral *Corallium*, one even named for that virtue, *Stylaster corallium* Cairns, 1986 [10].

Stylasterids and bryozoans can be remarkably convergent in their colony shape, so much so that four stylasterid species were originally described as bryozoans ([74]–[76], also see [77], and the reverse has also happened: two fossil species have been described as stylasterids that ultimately proved to be bryozoans [78], [79]).

### Future work

An obvious direction for future work with stylasterids would be an investigation into which molecular markers, mitochondrial and/or nuclear, are effective at discriminating species and genera, in an effort to validate or falsify the morphology-based species, and to construct a better phylogeny of the family. Thus far only one molecular study has been published with focus on the Stylasteridae]), which has used the following genes: mt16S, nuclear CaM (calmodulin), and nuclear18S [35]. In addition, two more general studies on hydrozoans have obtained nuclear 28S sequences from stylasterid species [80], [81]. Progress in this realm may be slow, as many species and even some genera have been collected only once from an exotic deep-water locality, and recollection of fresh material may prove to be problematic. Another need is to continue taxonomic revisions and the description of new species. In collections already made in deep water off New Caledonia by the MNHNP over 30 new species have been documented but not yet described [35], [82]. Equally high diversity might be expected from thorough collecting of the deep-water environments of the Philippines and Indonesia. The current number of 247 species might well double before we know the entire diversity of this fascinating group.
